# Editorial: High-tech personalized healthcare in movement disorders

**DOI:** 10.3389/fneur.2024.1452612

**Published:** 2024-07-02

**Authors:** Alessandro Zampogna, Luigi Borzì, Carolina Soares, Florenc Demrozi

**Affiliations:** ^1^Department of Human Neuroscience, Sapienza University of Rome, Rome, Italy; ^2^IRCCS Neuromed Institute, Pozzilli, Italy; ^3^Department of Control and Computer Engineering, Politecnico di Torino, Turin, Italy; ^4^Department of Neurology, Unidade Local de Saúde São João, E.P.E, Porto, Portugal; ^5^Department of Clinical Neurosciences and Mental Health, Faculty of Medicine of University of Porto, Alameda Prof. Hernâni Monteiro, Porto, Portugal; ^6^Department of Electrical Engineering and Computer Science, University of Stavanger, Stavanger, Norway

**Keywords:** movement disorders, Parkinson's disease, medical technology, telemedicine, wearable sensors, personalized medicine, machine learning

The clinical management of patients affected by movement disorders is rapidly evolving, driven by innovative health technologies and advanced computational techniques, such as wearable sensors, augmented reality tools, telemedicine systems, and artificial intelligence (AI) (1, 2). Technological advances offer new methods for early diagnosis, remote monitoring, tailored treatments, and enhanced rehabilitative strategies, all aimed at addressing individual needs through personalized approaches and increasing patients' quality of life. The opportunity to use these instruments directly at patients' homes allows for further improving therapeutic strategies by gathering ecological data recorded in free-living situations (3). In this context, new health technologies and computational techniques are promising tools possibly helpful for the overall clinical management of patients with movement disorders.

The present Research Topic entitled “High-Tech Personalized Healthcare in Movement Disorders” explores advances and perspectives of new technologies and AI-based analytical methods to support the clinical assessment as well as the therapeutic and rehabilitative management of patients suffering from movement disorders through objective methods. The ten manuscripts included in this Research Topic deal with the practical clinical application of various technologies and computational tools for the evaluation and treatment of a wide range of motor symptoms, such as gait disturbances and falls, upper limb impairment, tremor, and dysarthria, in patients affected by movement disorders. Accordingly, the articles in this Research Topic offer a comprehensive overview of healthcare technologies and computational solutions for clinical decision support in movement disorders.

Some studies within this Research Topic have focused on validating the employed tools to establish recording and analytical frameworks essential for managing the substantial volume of data generated by health technologies. For instance, Romijnders et al., from the Mobilise-D Consortium, conducted tests to validate an *ad hoc* developed deep learning algorithm for gait event detection in ecological environments. This involved utilizing pressure insoles and inertial measurement units (IMUs) in a broad sample of subjects with various neurological mobility-limiting diseases. Similarly, Russell et al. integrated measurements from IMUs with those from a microphone to explore the feasibility of measuring and predicting specific motor tasks using multimodal intent-sensing technology and innovative algorithms in patients with Parkinson's disease (PD).

Other researchers have explored the potential to improve the sensitivity of subjective patient assessment by integrating technological tools with conventional clinical instruments during real-time routine activities. In this context, Sena et al. examined the real-time mobility of patients hospitalized in intensive care units using wrist-worn accelerometers and deep-learning algorithms. Their findings highlighted the superior predictive accuracy of inertial measures combined with clinical data compared to traditional clinical scoring systems for assessing acuity in critical care settings.

Various authors have explored how technology and advanced analytical methods can be used to enhance telemonitoring and therapeutic approaches in movement disorders. van den Bergh et al. conducted remote monitoring of PD patients directly in their homes for a duration of 6 weeks, employing a sensor necklace and a smartphone app. They assessed the usability and effectiveness of the remote monitoring system in supporting physiotherapy interventions aimed at enhancing physical activity and preventing falls. Similarly, Sigcha et al. introduced a novel telemonitoring system named “Monipar,” designed for remote assessment of PD patients over extended periods. This system utilized accelerometers from off-the-shelf smartwatches and smartphone interfaces to quantitatively evaluate tremor and bradykinesia by guiding patients through standardized motor tasks. In another study, Suppa et al. showcased the feasibility of objectively assessing the impact of different therapeutic interventions, such as pharmacological treatment with L-Dopa and surgical therapy with sub-thalamic deep brain stimulation (DBS), on voice characteristics in PD patients. They employed advanced AI algorithms to also establish significant clinical-behavioral correlations between objective measures and qualitative clinical assessments of voice impairment. Hoogendoorn et al. investigated the effects of various wearable and flexible cueing techniques, including real-world or augmented reality cues, on gait performance in PD patients. Their findings demonstrated the efficacy of these systems in improving patients' walking abilities by directly influencing spatiotemporal gait parameters.

Finally, the potential of health technologies to enhance current remote rehabilitation strategies and teleneurology was also investigated in some studies included in this Research Topic. Vismara et al. applied exergames in a virtual environment to promote upper limb mobility in patients with movement impairment following a stroke. In the same way, Hardeman et al. employed home-based exergaming to improve gait, and balance, and reduce the risk of falls in PD patients, introducing an innovative approach using augmented reality glasses. Additionally, in a brief research report, Wan et al. explored a significant opportunity presented by new telemedicine approaches, which involves the remote programming of implantable pulse generators via the Internet for PD patients treated with DBS. The authors demonstrated that this approach is not only as effective as traditional in-person methods but also more cost-effective, offering several managerial advantages.

In conclusion, the achievements presented in this Research Topic underscore the pivotal role of technological advancements in reshaping clinical practice and improving outcomes for patients with movement disorders. These findings enable us to confidently affirm that, in the near future, new health technologies and advanced computational techniques will significantly contribute to the clinical management of these patients, underpinning a new “high-tech neurology” paradigm.

## Author contributions

AZ: Writing – original draft, Writing – review & editing. LB: Writing – review & editing. CS: Writing – review & editing. FD: Writing – review & editing.

## References

[1] Zampogna A Mileti I Palermo E Celletti C Paoloni M Manoni A. Fifteen years of wireless sensors for balance assessment in neurological disorders. Sensors. (2020) 20:3247. 10.3390/s20113247 32517315 PMC7308812

[2] Castelli Gattinara Di Zubiena F Menna G Mileti I Zampogna A Asci F Paoloni M. Machine learning and wearable sensors for the early detection of balance disorders in Parkinson's disease. Sensors. (2022) 22:9903. 10.3390/s22249903 36560278 PMC9782434

[3] Zampogna A Borzì L Rinaldi D Artusi CA Imbalzano G Patera M. Unveiling the unpredictable in Parkinson's disease: sensor-based monitoring of dyskinesias and freezing of gait in daily life. Bioengineering. (2024) 11:440. 10.3390/bioengineering11050440 38790307 PMC11117481

